# CT-based radiomics scores predict response to neoadjuvant chemotherapy and survival in patients with gastric cancer

**DOI:** 10.1186/s12885-020-06970-7

**Published:** 2020-05-25

**Authors:** Kai-Yu Sun, Hang-Tong Hu, Shu-Ling Chen, Jin-Ning Ye, Guang-Hua Li, Li-Da Chen, Jian-Jun Peng, Shi-Ting Feng, Yu-Jie Yuan, Xun Hou, Hui Wu, Xin Li, Ting-Fan Wu, Wei Wang, Jian-Bo Xu

**Affiliations:** 1 Department of Gastrointestinal Surgery, The First Affiliated Hospital of Sun Yat-Sen University, 58 Zhongshan Road 2, Guangzhou, 510080 People’s Republic of China; 2 Department of Medical Ultrasonics, Institute of Diagnostic and Interventional Ultrasound, The First Affiliated Hospital of Sun Yat-Sen University, 58 Zhongshan Road 2, Guangzhou, 510080 People’s Republic of China; 3 Department of Radiology, The First Affiliated Hospital of Sun Yat-sen University, Guangzhou, 510080 China; 4 Research Center of GE Healthcare, Shanghai, 200000 China

**Keywords:** Stomach neoplasms, Neoadjuvant therapy, Tomography, X-ray computed

## Abstract

**Background:**

Neoadjuvant chemotherapy is a promising treatment option for potential resectable gastric cancer, but patients’ responses vary. We aimed to develop and validate a radiomics score (rad_score) to predict treatment response to neoadjuvant chemotherapy and to investigate its efficacy in survival stratification.

**Methods:**

A total of 106 patients with neoadjuvant chemotherapy before gastrectomy were included (training cohort: *n* = 74; validation cohort: *n* = 32). Radiomics features were extracted from the pre-treatment portal venous-phase CT. After feature reduction, a rad_score was established by Randomised Tree algorithm. A rad_clinical_score was constructed by integrating the rad_score with clinical variables, so was a clinical score by clinical variables only. The three scores were validated regarding their discrimination and clinical usefulness. The patients were stratified into two groups according to the score thresholds (updated with post-operative clinical variables), and their survivals were compared.

**Results:**

In the validation cohort, the rad_score demonstrated a good predicting performance in treatment response to the neoadjuvant chemotherapy (AUC [95% CI] =0.82 [0.67, 0.98]), which was better than the clinical score (based on pre-operative clinical variables) without significant difference (0.62 [0.42, 0.83], *P* = 0.09). The rad_clinical_score could not further improve the performance of the rad_score (0.70 [0.51, 0.88], *P* = 0.16). Based on the thresholds of these scores, the high-score groups all achieved better survivals than the low-score groups in the whole cohort (all *P* < 0.001).

**Conclusion:**

The rad_score that we developed was effective in predicting treatment response to neoadjuvant chemotherapy and in stratifying patients with gastric cancer into different survival groups. Our proposed strategy is useful for individualised treatment planning.

## Background

Gastric cancer remains the third most frequent cause of cancer-related death worldwide, resulting in 782,685 deaths annually [1]. Despite the improvement in screening, a large proportion of patients in China are diagnosed at advanced stage. For locally advanced cases, the 5-year survival rate ranged from 20 to 30% after curative resection [2–4].

Given this poor prognosis, neoadjuvant chemotherapy has been tried for this patient population in recent years. After the promising results obtained with “MAGIC Trial”, “FFCD Trial”, “ACCORD Trial”, and “AIO-FLOT3 Trial”, neoadjuvant chemotherapy has become a promising treatment option for potentially resectable or limited metastatic gastric cancer with the improved 5-year survival rates of more than 35% [5–10]. Despite the satisfactory efficacy of neoadjuvant chemotherapy, patients’ responses varied between 30 and 60% [11]. A good response to neoadjuvant chemotherapy was associated with good survival outcome, while non-responding patients could suffer from adverse events and unnecessary costs and finally risk tumour progression and even miss the chance to undergo curative gastrectomy. Moreover, patients who are non-responsive to neoadjuvant chemotherapy could be waiting longer until surgery, and this extended time to surgery may be correlated with poorer survival of gastric cancer. Thus, early detection of those patients who are most likely to respond to neoadjuvant treatment is critical to provide them a chance for a timely surgery and to optimise the treatment plans. However, the treatment efficacy of neoadjuvant chemotherapy can only be assessed after three cycles of treatment. Therefore, exploring the pre-treatment predictors of treatment efficacy is important to determine the need for neoadjuvant therapy and the optimal timing for surgical resection, thus improving pre-treatment decision making.

Previous studies have investigated several imaging modalities such as contrast enhanced ultrasound, computed tomography (CT), magnetic resonance imaging, and positron emission tomography in the evaluation of patients’ response to chemotherapy for gastric cancer; however conflicting results were obtained [12–18]. Additionally, in these studies, analyses were only based on imaging features extracted by naked eyes or quantitative imaging parameters, and lacked a proper validation. Although naked eyes provide valuable feature information, some microcosmic imaging features relevant for clinical outcomes might be lost due to the limited visual image grey scales that can be detected by naked eyes. Radiomics is a rapidly growing discipline based on high-throughput quantitative image analysis to characterise tumours and their microenvironment. This approach can extract far more features than manual extraction by acquiring two-dimensional and high-dimensional imaging features using computer algorithm [19]. Many studies on other cancer types showed that radiomics features, such as texture features, filter transformed features, wavelet features, and so on, could not be visually observed but were closely related to pathologic microscopic structures and were effective in prognostic prediction [20–23].

Computed tomography is the preferred imaging examination for gastric cancer in clinical practice, but no literature has been reported on the application of CT-based radiomics technique to predict the response to neoadjuvant chemotherapy in gastric cancer patients. Therefore, we aimed to develop and validate a CT-based radiomics score to predict the response to neoadjuvant chemotherapy and stratify the survival for patients with gastric cancer.

## Methods

### Patients

**Fig. 1:**
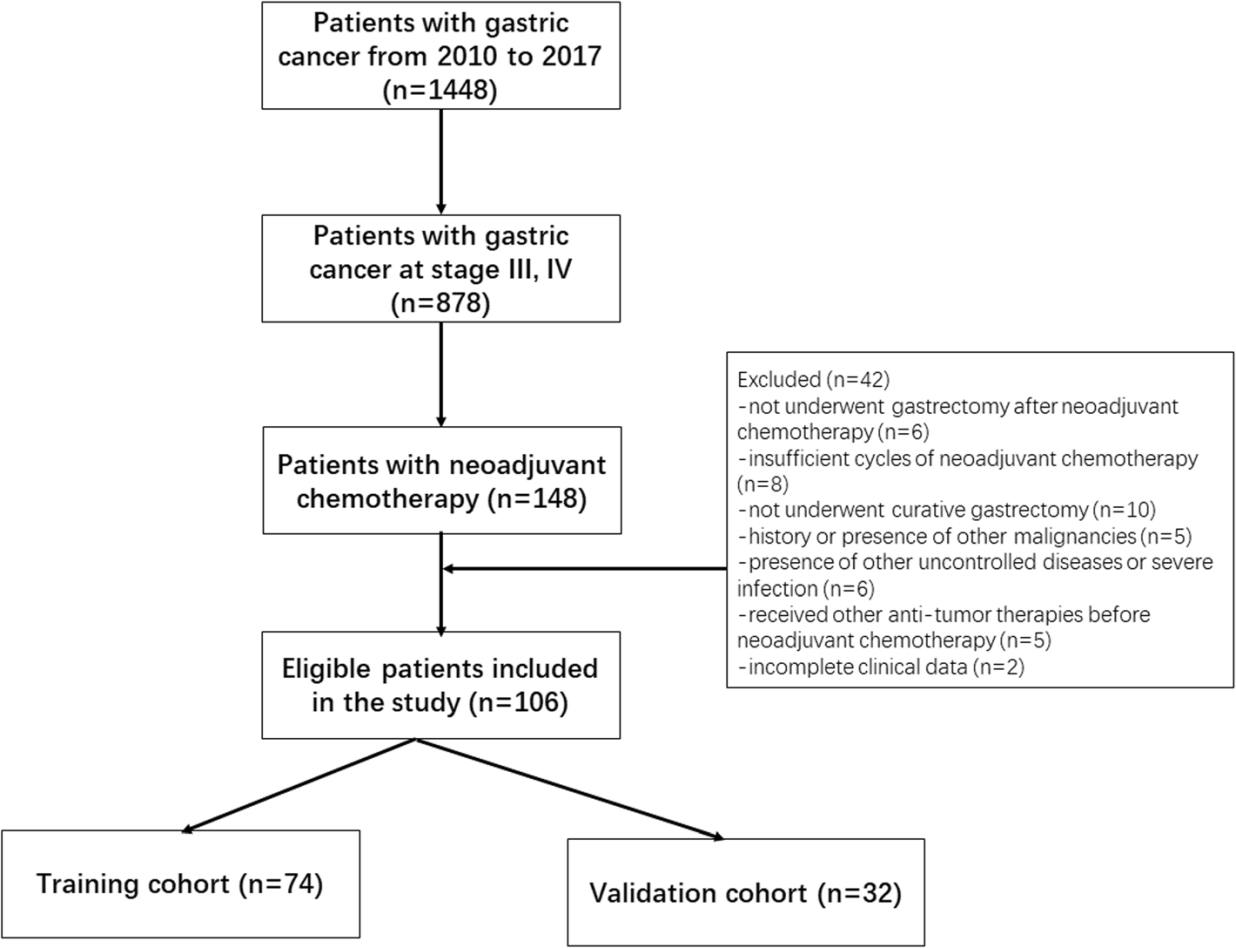
Flow diagram of study population

Consecutive patients diagnosed with gastric cancer between January 2010 and December 2017 were identified by reviewing the database of the Center of Gastrointestinal Surgery of the First Affiliated Hospital of Sun Yat-Sen University. Patients were included according to the following criteria: (1) histologically confirmed gastric adenocarcinoma on gastroscopy; (2) potential resectable gastric cancer at clinical stage of III, IV as determined by pretreatment contrast-enhanced CT (patients with M1 were those with only para-aortic lymph node metastasis without any other risk of curative resection); (3) received neoadjuvant chemotherapy of SOX regimen (S-1 plus oxaliplatin) as the initial treatment; (4) underwent curative gastrectomy; (5) received contrast-enhanced CT within one week before neoadjuvant chemotherapy; (6) Eastern Cooperative Oncology Group performance status between 0 to 1; (7) a life expectancy of > 3 months; (8) adequate bone marrow, renal, and hepatic function [platelets > 80 × 10^9^/L, absolute neutrophil count ≥1.5 × 10^9^/L, serum creatinine≤1.5 mg/dL, total bilirubin level within 1.5 × the upper limit of normal (ULN), and serum transaminase ≤2.5× ULN]. The following exclusion criteria were used: (1) history or presence of other malignancies; (2) presence of other uncontrolled diseases or severe infection; (3) received other anti-tumour therapies before neoadjuvant chemotherapy; (4) incomplete clinical data. The patient selection process is shown in Fig. 1. Patients were randomly allocated to the training and validation cohorts at the ratio of 7:3. Our Institutional Ethic Review Board has approved the current study, following the regulations outlined in the Declaration of Helsinki.

### Neoadjuvant chemotherapy

Patients received the first-line neoadjuvant chemotherapy of SOX regimen. S-1 was orally administered twice daily at concentrations based on body surface area (BSA): BSA < 1.25 m^2^, 80 mg/d; 1.25 m^2^ BSA < 1.50 m^2^, 100 mg/d; and BSA ≥ 1.50, 120 mg/d. On the first day, oxaliplatin (130 mg/m^2^) was administered via intravenous infusion, followed by S-1 administered for 14 consecutive days, followed by a 1-week break for a maximum of three cycles, until tumour progression, presence of unacceptable toxicity or treatment withdrawal by the patient or doctor.

### Assessment of the response to neoadjuvant chemotherapy

The treatment response to neoadjuvant chemotherapy was evaluated via pathologic response. Haematoxylin and eosin-stained slides were reviewed by two pathologists with more than 10 years of experience in gastrointestinal pathology who were blinded to the clinical data, and they graded the specimens for pathologic response according to the Mandard tumour regression grading (TRG) system [24]. TRG 1 was defined as complete regression/fibrosis with no viable tumour cells, TRG 2 was defined as fibrosis with scattered tumour cells, TRG 3 was defined as fibrosis and tumour cells with predominant fibrosis, TRG 4 was defined as fibrosis and tumour cells with predominant tumour cells, and TRG 5 was defined as tumour without evidence of regression. Disagreement was resolved by discussion with consensus. Responders were defined as TRG 1–2 and non-responders were defined as TRG 3–5 [25].

### CT images acquisition

**Fig. 2:**
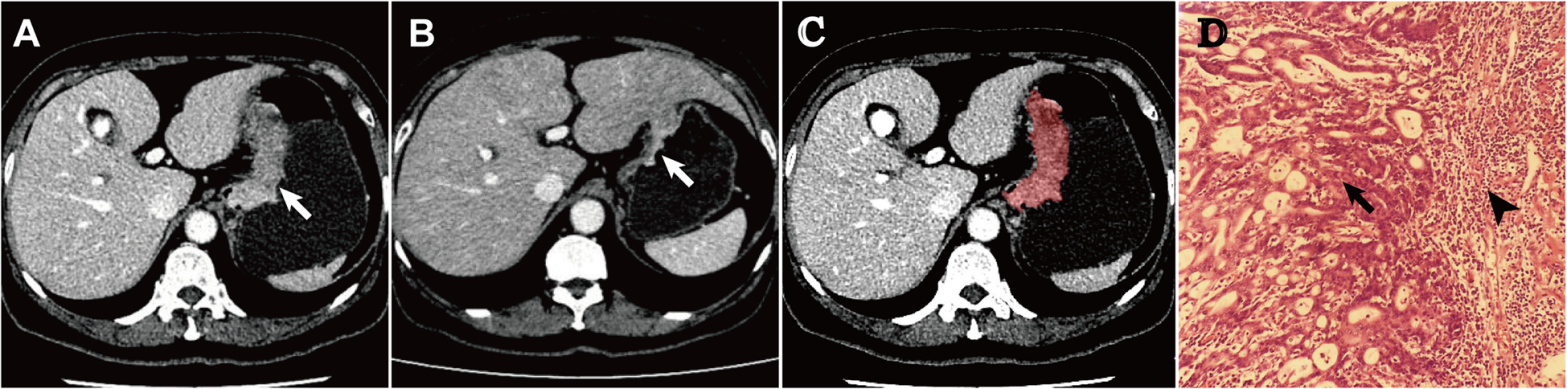
A female patient was diagnosed as gastric cancer (T4aN2M0). CT before neoadjuvant chemotherapy (**a**) showed a mass-type tumor measured 25 mm in maximal depth and 80 mm in maximal length. CT after neoadjuvant chemotherapy (**b**) showed a shrunken mass measured 14 mm in depth and 40 mm in length. CT before neoadjuvant chemotherapy (**c**) showed the ROI delineated manually on figure (**a**). Pathology examination after surgery (**d**) showed residual tumor tissue (arrow) and infiltrated inflammatory cells (arrow head)

The standard dynamic contrast-enhanced MDCT scan (Aquilion 64; Toshiba Medical System, Tokyo, Japan) procedure was used. Briefly, after an unenhanced helical sequence scan from the liver dome to the symphysis pubis, venous phase contrast-enhanced CT was performed after a 65-s delay following intravenous administration of 80–100 ml (1.5 ml/kg) of iodinated contrast agent (Ultravist 300; Schering, Berlin, Germany) administered via the antecubital vein at a rate of 2.0–3.0 ml/s. The following CT acquisition parameters were used: 120 kV, 200–250 mAs, rotation time of 0.5 s, collimation of 64 mm × 0.5 mm, slice thickness of 0.5 mm, slice increments of 0.5 mm, pitch of 0.9, field of view of 350 × 350 mm, matrix of 512 × 512, and reconstruction thickness of 2.5 mm. CT images were retrieved from the picture archiving and communication system (PACS) (HP workstation XW8200, VitreaCore, version 3.7) for image analysis. The display window width was 150–350 HU, and the window level was 50 to 80 HU. One such case is presented in Fig. 2 with CT images before and after the neoadjuvant chemotherapy and the image of response assessment by pathology.

### Radiomics feature extraction

Portal venous phase contrast-enhanced CT images were used for radiomics feature extraction because of the better differentiation between the tumour tissue and the adjacent normal tissue of the gastric wall in the portal venous phase than in arterial phase. A region of interest (ROI) was delineated around the tumour outline for the largest cross-sectional area while excluding the air area by two independent radiologists with more than five years of experience in gastrointestinal imaging, and any disagreements were resolved by the consensus with arbitration by a third author. For each ROI, a total of 1044 imaging features were extracted and analyzed by an in house-made software: the A.K. software (Analysis-Kit, version 2.0.0, GE healthcare), which included six kinds of features (Supplemental Table 1): 42 histogram parameters, 10 texture parameters, 9 form factor parameters, 432 grey level co-occurrence matrix (GLCM), 540 grey level run-length matrix (GLRLM), and 11 grey level Size Zone Matrix (GLSZM).

### Feature reduction and model building

The included patients were divided into the training and validation cohorts by a ratio of 7:3 using random-stratified grouping. In the training cohort, support vector machine (SVM) and principle component analysis (PCA) were used to select significant radiomics features in the tumour associated with patient response to neoadjuvant chemotherapy [26, 27]. Based on the selected radiomics features, the Extremely Randomised Tree (Extra-Trees) method was applied to construct the radiomics score (rad_score) [28, 29]. The detailed Extra-Trees method is described in the Supplemental Materials. Then, the clinical variables were selected for the univariable and multivariable logistic regression models based on the backward selection with *P*-values less than 0.05 in the training cohort. A clinical score was formulated based on the clinical variables selected from the multivariable model. The significant clinical variables and radiomics score were integrated to establish the rad_clinical_score.

### Model evaluation and comparison

All the three scores were applied to classify responders and non-responders to neoadjuvant chemotherapy, and the results were validated in the validation cohort. The diagnostic ability of these scores was assessed with the area under the characteristics operating curves (AUC), accuracy, sensitivity, specificity, positive predictive value, and negative predictive value. The comparisons of these scores in predicting responders to neoadjuvant chemotherapy were performed using the AUCs and decision curve analysis (DCA). DCA was conducted to determine the clinical usefulness of these scores by quantifying the net benefits at different threshold probabilities.

### Survival analysis

In the whole cohort, the clinical score and rad_clinical_score were updated with post-operative clinical variables. Univariable and multivariable Cox regression analyses were performed to investigate the prognostic effects of rad_score, updated clinical score, and rad_clinical_score. According to the thresholds obtained when the Youden index was the largest, patients were stratified into high-score and low-score groups respectively by the above three scores. Kaplan-Meier curves were plotted and survival rates were compared between two groups using log-rank tests.

### Statistical analyses

The feature reduction and model building were performed in Python (version 2.7.14), utilising ExtraTreesClassifier from Scikit-learn. Other statistical analyses were performed by R software version 3.2.3 (R Foundation for Statistical Computing, Vienna, Austria, https://www.R-project.org/). The continuous variables were presented as mean ± standard deviation or median and quartile, and the categorical variables were presented as frequencies and percentage. Independent sample *t*-test or Kruskal-Wallis (KW) nonparametric rank sum test was used to compare the baseline characteristics between the training and validation cohorts, and between responding group and non-responding group for continuous variables, while Chi-square test or Fisher exact test for categorical variables. A two-sided *P*-value was considered statistically significant if less than 0.05.

## Results

### Baseline characteristics

A total of 106 patients were included, with 74 patients in the training cohort and 32 in the validation cohort. These two cohorts were comparable in baseline characteristics (Table 1). The median time interval between the surgery and chemotherapy was 73 days (range, 70–77 days) in the training cohort and 74 days (range, 70–77) in the validation cohort.

**Table 1:** Clinicopathological characteristics of the training and validation cohorts

Factors	Training cohort (***n*** = 74)	Validation cohort (***n*** = 32)	***P*** value
Age (years, mean ± SD)	55.15 ± 11.43	54.13 ± 12.68	0.68
Gender			0.53
Male	48	18	
Female	26	14	
BMI (kg/m^2^)	21 ± 3	22 ± 3	0.12
Preoperative T stage			0.39
2	1	0	
3	11	2	
4	62	29	
Preoperative N status			0.99
0	2	1	
1–3	72	31	
Preoperative M status			0.85
0	47	19	
1	27	13	
Postoperative T stage			0.83
1–2	11	6	
3–4	63	26	
Postoperative N status			0.06
0	35	8	
1–3	39	24	
Postoperative M status			0.90
0	53	24	
1	21	8	
Postoperative TNM stage			0.23
0	3	3	
1	6	1	
2	22	5	
3	22	15	
4	21	8	
AFP (ng/mL)	9.25 ± 17.79	6.48 ± 8.33	0.40
CEA (IU/L)			0.23
Normal	50	26	
Elevated	24	6	
CA125 (IU/L)			0.08
Normal	73	29	
Elevated	1	3	
CA199 (IU/L)			1.00
Normal	64	27	
Elevated	10	5	
Operative duration (min)	349.46 ± 116.47	359.22 ± 111.98	0.69
Blood transfusion (ml)	347.92 ± 506.39	362.50 ± 458.43	0.89
Total number of dissected lymph node	40.81 ± 18.67	44.09 ± 17.77	0.40
Number of positive lymph node	5.27 ± 9.01	7.34 ± 10.42	0.30
Treatment response			0.25
TRG 1	3	3	
TRG 2	34	14	
TRG 3	24	10	
TRG 4	10	4	
TRG 5	3	1	

*Abbreviations*: *BMI* body mass index, *PS* performance status, *AFP* alpha-fetoprotein, *CEA* carcinoembryonic antigen, *TRG* tumor regression grading

### Model construction

In the training cohort, SVM and PCA analysis identified 25 radiomics features significantly associated with the response to neoadjuvant chemotherapy. These features were histogram parameters, GLCM, and GLRLM, with GLRLM accounting for the majority (Supplemental Table 2). A rad_score was established based on the above 25 radiomics features using Extra-Trees method. Age and preoperative M status were found to be significantly different between responding group and non-responding group (both *P* < 0.05) (Table 2), and thus a clinical score was built based on them. By integrating the rad_score and two clinical variables, a rad_clinical_score was derived using SVM algorithm. Results showed that the rad_score (Odds ratio [OR] = 1.21 × 10^5^, 95% confidence interval [CI]: 52.3–3.07 × 10^9^, *P* < 0.01) was significantly associated with the treatment response of neoadjuvant chemotherapy (Table 3), and the rad_clinical_score was marginally associated with treatment response (*P* = 0.06), whereas the clinical score was not (*P* = 0.28).

**Table 2:** Comparison of clinical variables and radiomics score in the responding group and non-responding group in the training cohort

Factors	Responding group	Non-responding group	***P*** value
Age (years, mean ± SD)	56.76 ± 11.42	52.85 ± 11.91	0.02
Gender			1.00
Male	24	24	
Female	13	13	
BMI (kg/m^2^)	21 ± 3	21 ± 4	0.42
AFP (ng/mL)	8.95 ± 13.51	7.86 ± 17.55	0.56
CEA (IU/L)			0.80
Normal	26	24	
Elevated	11	13	
CA199 (IU/L)			1.00
Normal	32	32	
Elevated	5	5	
CA125 (IU/L)			1.00
Normal	36	37	
Elevated	1	0	
Preoperative T stage			0.17
2	0	1	
3	8	3	
4	29	33	
Preoperative N status			0.19
0	2	0	
1	13	8	
2	18	21	
3	4	8	
Preoperative M status			0.05
0	28	19	
1	9	18	
Radiomics score	0.54 ± 0.22	0.41 ± 0.22	< 0.01

*Abbreviations*: *BMI* body mass index, *PS* performance status, *AFP* alpha-fetoprotein, *CEA* carcinoembryonic antigen

**Table 3:** Association of the three scores with treatment response of neoadjuvant chemotherapy for gastric cancer

Cohorts	Models	Responding group	Non-responding group	OR (95% CI)	*P* value
Training cohort	rad_score	0.56 ± 0.26	0.38 ± 0.25	14.51 (2.40, 98.35)	< 0.01
	clinical_score	0.56 ± 0.11	0.47 ± 0.13	355.62 (7.98, 2.41*10^4^)	< 0.01
	rad_clinical score	−0.61 ± 0.29	−0.88 ± 0.34	12.22 (2.79, 64.65)	< 0.01
Validation cohort	rad_score	0.54 ± 0.12	0.42 ± 0.08	1.21*10^5^ (52.25, 3.07*10^9^)	< 0.01
	clinical_score	0.52 ± 0.12	0.48 ± 0.11	33.46 (0.07, 2.98*10^4^)	0.28
	rad_clinical score	−0.38 ± 0.23	−0.56 ± 0.27	16.90 (1.04, 422.82)	0.06

*Abbreviations*: *OR* odds ratio, *CI* confidence interval

### Model performance in response prediction and validation

**Fig. 3:**
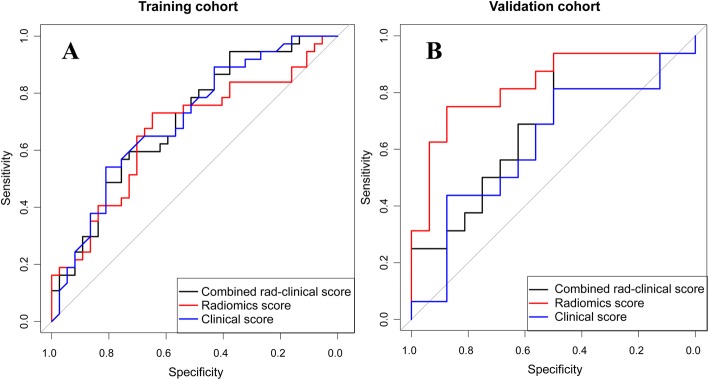
Receiver operating characteristics curves of the three scores in the training and validation cohorts. **a** in the training cohort; **b** in the validation cohort

**Fig. 4:**
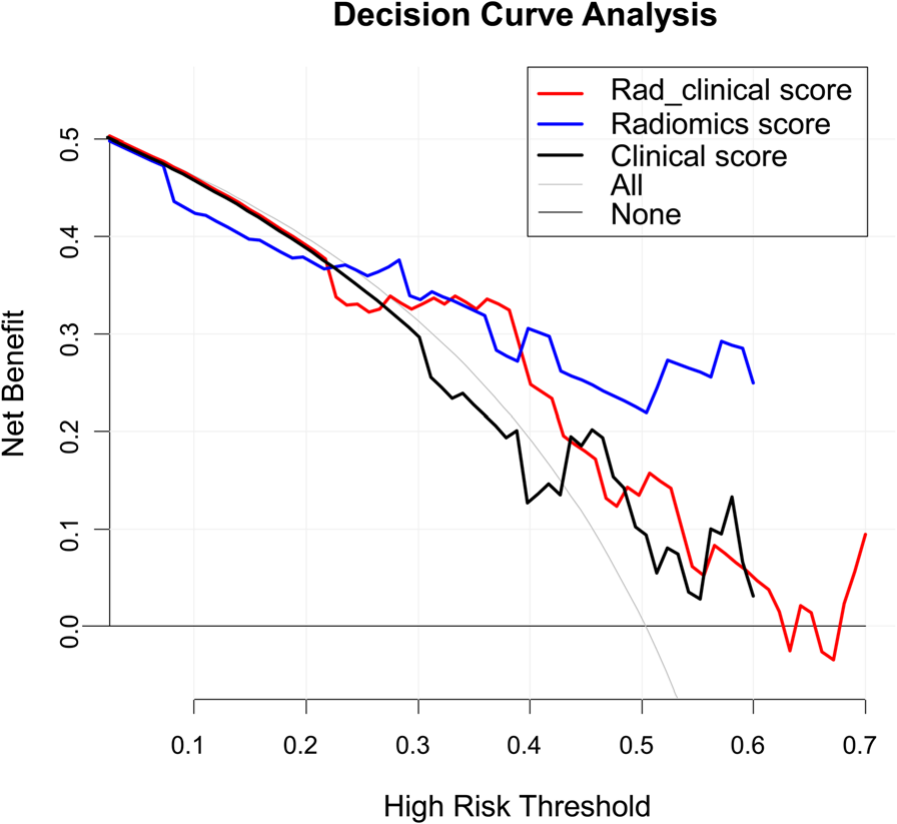
Decision curve analysis for the rad_score, clinical score and rad_clinical score

The rad_score was effective in predicting responders to neoadjuvant chemotherapy in the training cohort (AUC: 0.77, 95% CI: 0.65–0.88) and in the validation cohort (AUC: 0.82, 95% CI: 0.67–0.98) (Fig. 3). Compared to the rad_score, the clinical score was poorer in predicting accuracy without significant difference (training: 0.70, 95% CI: 0.58–0.82, *P* = 0.15; validation: 0.62, 95% CI: 0.42–0.83, *P* = 0.09), and the rad_clinical_score did not demonstrate an improved performance (training: 0.70, 95% CI: 0.58–0.82, *P* = 0.12; validation: 0.70, 95% CI: 0.51–0.88, *P* = 0.16) (Fig. 3). The DCA showed that the rad_score had the higher overall net benefit compared with the rad_clinical_score and clinical score across the majority of the risk of responders (Fig. 4). Other detailed predicting performance is described in Table 4.

**Table 4:** Predictive performance of the three scores in the treatment response of neoadjuvant chemotherapy for gastric cancer in the validation cohort

	Cut-off	ACC		SEN		SPE		PPV		NPV	
	–	ACC	*P*	SEN	*P*	SPE	*P*	PPV	*P*	NPV	*P*
rad_score	0.516	0.81	–	0.75	–	0.88	–	0.86	–	0.78	–
clinical_score	0.462	0.63	0.11	0.75	1.00	0.50	< 0.01	0.60	0.02	0.67	0.32
rad_clinical score	−0.651	0.69	0.26	0.88	0.18	0.50	< 0.01	0.64	0.04	0.80	0.84

*Abbreviations*: *ACC* accuracy, *SEN* sensitivity, *SPE* specificity, *PPV* positive predictive value, *NPV* negative predictive value

### Survival stratification by the models

**Fig. 5:**
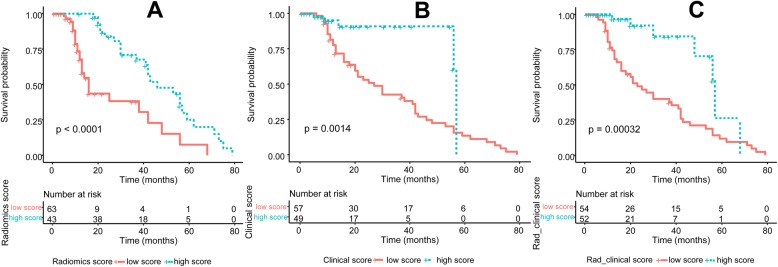
Comparisons of the overall survivals between high-score group and low-score group respectively stratified by rad_score, clinical score and rad_clinical score. **a** stratified by rad_score; **b** stratified by clinical score; **c** stratified by rad_clinical_score

In the whole cohort, univariable and multivariable Cox regression analyses showed that the rad_score (Hazard Ratio [HR] = 0.22, 95% CI: 0.11–0.42, *P* < 0.01) was significantly associated with OS (Table 5). Univariable analysis showed that preoperative T status (HR = 2.59, 95% CI: 1.03–6.53, *P* = 0.04), the total number of dissected lymph nodes (HR = 1.03, 95% CI: 1.00–1.06, *P* = 0.04), and postoperative N status (HR = 2.09, 95% CI: 1.48–3.98, *P* < 0.01) were significantly associated with OS. Based on these clinical variables, the clinical_score was updated and also found to be significantly associated with OS (HR = 2.65, 95% CI: 1.07–6.54, *P* = 0.03). Furthermore, the rad_clinical_score was also updated by integrating the rad_ score with the new selected clinical variables, and was found to be associated with OS (HR = 2.65, 95% CI: 1.07–6.54, *P* = 0.03). Based on the threshold of rad_score of 0.59, patients were divided into groups either with high-score or with low score. The OS in patients from the high-score group was significantly higher than that in patients from the low-score group (*P* < 0.001) (Fig. 5**a**). Similarly, the high-score groups stratified by the rad_clinical_score (*P* < 0.001) and clinical score (*P* < 0.001) both achieved longer OS than the low-score groups (Fig. 5b, c).

**Table 5:** Multivariable analysis of the three scores and clinicopathological characteristics with overall survival

Factors	HR	95% CI	***P*** value
Preoperative T stage	2.59	1.03–6.53	0.04
Total number of dissected lymph node	1.03	1.00–1.06	0.04
Postoperative N status	2.09	1.48–3.98	< 0.01
TNM stage	2.67	1.15–6.23	0.02
rad_score	0.22	0.11–0.42	< 0.01
clinical_score	2.65	1.07–6.54	0.03
rad_clinical_score	4.27	1.18–15.39	0.03

*Abbreviations*: *HR* hazard ratio, *CI* confidence interval

## Discussion

Our study constructed and validated an effective CT-radiomics score for predicting treatment response to neoadjuvant chemotherapy in patients with potentially resectable or limited metastatic gastric cancer. The rad_clinical_score which was derived by combining clinical variables with radiomics features, could not further improve the predicting performance when compared to the rad_score. Moreover, the rad_score was capable to stratify patients into two groups with different survival outcomes.

To the best of our knowledge, this is the first attempt we develop radiomics scores to predict the response to neoadjuvant chemotherapy in patients gastric cancer before treatment. Given the great therapeutic efficacy of neoadjuvant chemotherapy for responding patients and high risk of non-response in patients [11], the early identification of potentially responding patients who might benefit from neoadjuvant chemotherapy is important to maximise treatment efficacy and optimise personalised therapy. Our established rad_score performed well in this respect, indicating the possibility of radiomics in predicting treatment response of neoadjuvant chemotherapy for gastric cancer. Several studies were conducted previously on the texture or radiomics analysis in the evaluation of treatment response in gastric cancer. Jiang et al. developed a radiomics signature which was effective in predicting chemotherapy efficacy in patients with stage II and III gastric cancer [30]. Yoon et al. showed that texture features on CT images were correlated with the prognosis in patients with HER2-positive advanced gastric cancer who received trastuzumab-based treatment, with heterogeneous features suggestive of better survival outcomes [31]. Therefore, the underlying reason for our good model performance might be the fact that intratumoural heterogeneity reflected by radiomic features was associated with tumour biology and even cell cycle regulating pathways, which are strong factors influencing the efficacy of neoadjuvant chemotherapy [32–34]. The full mechanism behind the relationship between radiomic features and neoadjuvant chemotherapy has not been elucidated, and radiogenomics studies are warranted to provide evidence in this issue [35]. Besides, by integrating clinical variables with radiomics features, the derived rad_clinical_score could not show superior predicting performance to that of the rad_score. This indicated that radiomics features were the stronger component of this combined score while clinical data had limited impact in elevating the performance.

In addition, our rad_score was capable to stratify patients into two groups with different risks of death, which helped us identify the subgroup of patients with poor prognosis for whom more intensified treatment and closer follow-up schedule was needed. Low rad_score was associated with poor prognosis, which made sense because low rad_score was associated with no or poor response to neoadjuvant chemotherapy. It was reported that patients who responded to neoadjuvant chemotherapy had a higher likelihood to receive curative gastrectomy, and their survival was expected to be better than that of non-responding patients [5–9]. The finding that the rad_score developed using the outcome of treatment response to neoadjuvant chemotherapy was effective in prognosis stratification, further confirmed its clinical significance and usefulness. Instead of two models, our single model could be used in both the prediction of treatment response and survival stratification.

Previous studies have found that radiomics features were closely related to tumour biology and microscopic structure [36–39]. Our study identified 25 radiomic features associated with treatment response to neoadjuvant chemotherapy for gastric cancer. These were histogram parameters, GLCM, and GLRLM with more than half of the features being GLRLM. GLCM and GLRLM were important markers of intra-tumour homogeneity, because they represented the level of signal heterogeneity in a lesion in the manner of relative relationship between the distribution and site of the gray level. These values (GLCM and GLRLM) were higher in patients with no response to neoadjuvant chemotherapy, which indicated that the intratumoral heterogeneity was more apparent in these patients than in the responding patients. Many studies have reported that tumours with greater intratumoral heterogeneity tended to be more aggressive in terms of proliferation, metastasis, and angiogenesis [22, 40], and thus might be more resistant to neoadjuvant chemotherapy.

There are several limitations in our study. First, the sample size was small considering the relatively large number of variables. Therefore, Extremely Randomised Tree method was used to minimise the bias because it used the whole training sample rather than a bootstrap replica to build a tree, and it included a random subset of features and split nodes by choosing cut-points at random within each tree. Second, our models lacked the external validation, which reduced the confirmation strength of the model accuracy.

## Conclusion

The radiomics score developed in this study was effective in predicting treatment response to neoadjuvant chemotherapy and stratifying patients’ prognosis for gastric cancer. These findings may help clinicians in identifying potentially responding patients and providing personalised treatment.

## Supplementary information

**Additional file 1: Table S1.** A Summary of 1044 Radiomics Features, **Table S2.** A summary of radiomics features significantly associated with treatment response of neoadjuvant chemotherapy.

## Data Availability

Data would be available from the corresponding author on reasonable request.

## Abbreviations

CT: Computed tomography
MDCT: Multi-detector computed tomography
ULN: Upper limit of normal
BSA: Body surface area
TRG: Tumor regression grading
PACS: Picture archiving and communication system
ROI: Region of interest
GLCM: Grey level co-occurrence matrix
GLRLM: Grey level run-length matrix
GLSZM: Gray level Size Zone Matrix
SVM: Support vector machine
PCA: Principle component analysis
AUC: Area under the curve
DCA: Decision curve analysis
OS: Overall survival
