# Supplementary material for: CT-based radiomics scores predict response to neoadjuvant chemotherapy and survival in patients with gastric cancer
Source: BMC Cancer. 2020 May 25;20:468. doi: 10.1186/s12885-020-06970-7 (PMC7249312; doi:10.1186/s12885-020-06970-7)
Supplement: Supplementary file 1 — Additional file 1: Table S1. A Summary of 1044 Radiomics Features, Table S2. A summary of radiomics features significantly associated with treatment response of neoadjuvant chemotherapy. [file 12885_2020_6970_MOESM1_ESM.docx]

**Development and Validation of CT-based Radiomics Scores for Prediction of Response to Neoadjuvant Chemotherapy and Survival in Gastric Cancer**

Kaiyu Sun^1#^, Hangtong Hu^2#^, Shuling Chen^2^, Jinning Ye^1^, Guanghua Li^1^, Lida Chen^2^, Jianjun Peng^1^, Shiting Feng^3^, Yujie Yuan^1^, Xun Hou^1^, Xin Li^4^, Tingfan Wu^4^, Wei Wang^2^*, Jianbo Xu^1^*

***Corresponding authors**

Wei Wang, M.D., Ph.D.

Department of Medical Ultrasonics, Institute of Diagnostic and Interventional Ultrasound, The First Affiliated Hospital of Sun Yat-Sen University, 58 Zhongshan Road 2, Guangzhou, 510080, People’s Republic of China;

Phone and Fax: 86-20-87765183;

E-mail: [wangw73@mail.sysu.edu.cn](mailto:wangw73@mail.sysu.edu.cn).

Jianbo Xu, M.D., Ph.D.

Department of Gastrointestinal Surgery, The First Affiliated Hospital of Sun Yat-Sen University, 58 Zhongshan Road 2, Guangzhou, 510080, People’s Republic of China;

Phone and Fax: 86-20-87765183;

E-mail: [xjianb@mail.sysu.edu.cn](mailto:xjianb@mail.sysu.edu.cn).

**Supplemental Materials**

***Extremely Randomized Tree (Extra-Trees) method***

Unlike other tree-based ensemble methods, Extra-Trees algorithm uses the whole training sample rather than a bootstrap replica subset of features and splits nodes by choosing cut-points at random as well. The main parameters including the minimum number of samples for splitting a node, the number of trees of an ensemble, and the number of features per node for the Extra-Trees splitting procedure were set and adjusted by 5-fold cross-validation to create a predictive model of treatment response of neoadjuvant chemotherapy with the best performance in the training dataset. To create an optimal model, we coded a comprehensive computer program to train and evaluate hundreds of models, conducted extensive grid search in the hyper-parameter space through multiple combinations of parameter tunes, and tested different feature sets to enhance its accuracy and model performance.

**Supplementary Table 1. A Summary of 1044 Radiomics Features**

| **Features classes** | **Statistics** | **Count** |
| --- | --- | --- |
| **Histogram Parameters ^1^** | Mean, variance, median, min, max, percentile, quantile, expectation, skewness, kurtosis, energy, entropy, uniformity | 42 |
| **Texture Parameters ^2^** | Probability, energy, entropy, mean, expectation, variance | 10 |
| **Form Factor Parameters ^3^** | Sphericity, surface area, volume | 9 |
| **Grey level co-occurrence matrix (GLCM) ^4^** | Probability, energy, entropy, mean, expectation, Variance | 432 |
| **Grey level run-length matrix (GLRLM) ^5^** | Probability, energy, entropy, mean, expectation, Variance | 540 |
| **Gray level Size Zone Matrix (GLSZM) ^6^** | Probability, energy, entropy, mean, expectation, Variance | 11 |
| **Total** |  | 1044 |

1. Histogram parameters are concerned with properties of individual pixels. They describe the distribution of voxel intensities within the CT image through commonly used and basic metrics.
2. Texture represents the appearance of the surface and how its elements are distributed. In a sense it assists in predicting the feeling of the surface (e.g. smoothness, coarseness …etc.) from image. AK software is mainly concerned with texture classification accuracy improvement using textures features statistical based methods.
3. Form factor includes descriptors of the three-dimensional size and shape of the tumor region.
4. Grey level co-occurrence matrix (GLCM) P (*i, j*|*θ*, *d*) represents the joint probability of certain sets of pixels having certain grey-level values. It calculates how many times a pixel with grey-level *i* occurs jointly with another pixel having a grey value *j* by varying the displacement vector *d* between each pair of pixels. GLCM of an image is computed using displacement vector d defined by its radius, and rotational angles *θ*.
5. The grey level run-length matrix (GLRLM) P_r (*i*, *j* | *θ* ) is defined as the numbers of runs with pixels of gray level *i* and run length *j* for a given direction *θ*. RLMs is generated for each sample image segment having directions (0°, 45°, 90° & 135°).
6. The gray level Size Zone Matrix (GLSZM) is the starting point of Thibault matrices. For a texture image f with N gray levels, it is denoted GSf (*s*, *g*) and provides a statistical representation by the estimation of a bivariate conditional probability density function of the image distribution values. It is calculated according to the pioneering Run Length Matrix principle: the value of the matrix GSf (*s*, *g*) is equal to the number of zones of size *s* and of gray level *g*.

**Supplemental Table 2. A summary of radiomics features significantly associated with treatment response of neoadjuvant chemotherapy**

| MaxIntensity | uniformity | GLCMEntropy_angle45_offset1 | GLCMEntropy_angle90_offset1 |
| --- | --- | --- | --- |
| Inertia_angle0_offset3 | Inertia_angle0_offset4 | GLCMEntropy_angle135_offset4 | InverseDifferenceMoment_AllDirection_offset5_SD |
| GLCMEntropy_AllDirection_offset8_SD | GLCMEntropy_angle45_offset8 | Correlation_angle90_offset9 | HaralickCorrelation_angle90_offset9 |
| RunLengthNonuniformity_AllDirection_offset1_SD | LongRunLowGreyLevelEmphasis_AllDirection_offset1_SD | LongRunEmphasis_angle90_offset1 | ShortRunLowGreyLevelEmphasis_AllDirection_offset2_SD |
| RunLengthNonuniformity_AllDirection_offset1_SD | LongRunLowGreyLevelEmphasis_AllDirection_offset1_SD | LongRunEmphasis_angle90_offset1 | ShortRunLowGreyLevelEmphasis_AllDirection_offset2_SD |
| RunLengthNonuniformity_AllDirection_offset1_SD | LongRunLowGreyLevelEmphasis_AllDirection_offset1_SD | LongRunEmphasis_angle90_offset1 | ShortRunLowGreyLevelEmphasis_AllDirection_offset2_SD |
| ShortRunLowGreyLevelEmphasis_AllDirection_offset9_SD |  |  |  |
